# Rapid Sampling of Large Quantities of Interstitial Fluid from Human Skin Using Microneedles and a Vacuum-assisted Skin Patch

**DOI:** 10.21769/BioProtoc.5173

**Published:** 2025-02-05

**Authors:** Elizabeth C. Wilkirson, Xue Jiang, Peter B. Lillehoj

**Affiliations:** 1 Department of Mechanical Engineering, Rice University, Houston, TX, USA; 2 Spear Bio, Woburn, MA, USA; 3 Department of Bioengineering, Rice University, Houston, TX, USA

**Keywords:** Microneedle, Interstitial fluid, Skin, Dermal, Biomarker

## Abstract

Interstitial fluid (ISF) is a promising diagnostic sample due to its extensive biomolecular content while being safer and less invasive to collect than blood. However, existing ISF sampling methods are time-consuming, require specialized equipment, and yield small amounts of fluid (<5 μL). We have recently reported a simple and minimally invasive technique for rapidly sampling larger quantities of dermal ISF using a microneedle (MN) array to generate micropores in the skin from which ISF is extracted using a vacuum-assisted skin patch. Here, we present step-by-step protocols for fabricating the MN array and skin patch, as well as for using them to sample ISF from human skin. Using this technique, an average of 20.8 μL of dermal ISF can be collected within 25 min, which is a ∼6-fold improvement over existing ISF sampling methods. Furthermore, the technique is well-tolerated and does not require the use of expensive or specialized equipment. The ability to collect ample volumes of ISF in a quick and minimally invasive manner will facilitate the analysis of ISF for biomarker discovery and its use for diagnostic testing.

Key features

• Minimally invasive (bloodless and nearly painless) technique for sampling ISF from human skin.

• An average of 20.8 μL of interstitial fluid can be collected within 25 min.

• This technique does not require expensive or specialized equipment or electricity.

• Collected ISF can be analyzed using conventional laboratory-based assays or point-of-care diagnostic tests.

## Graphical overview


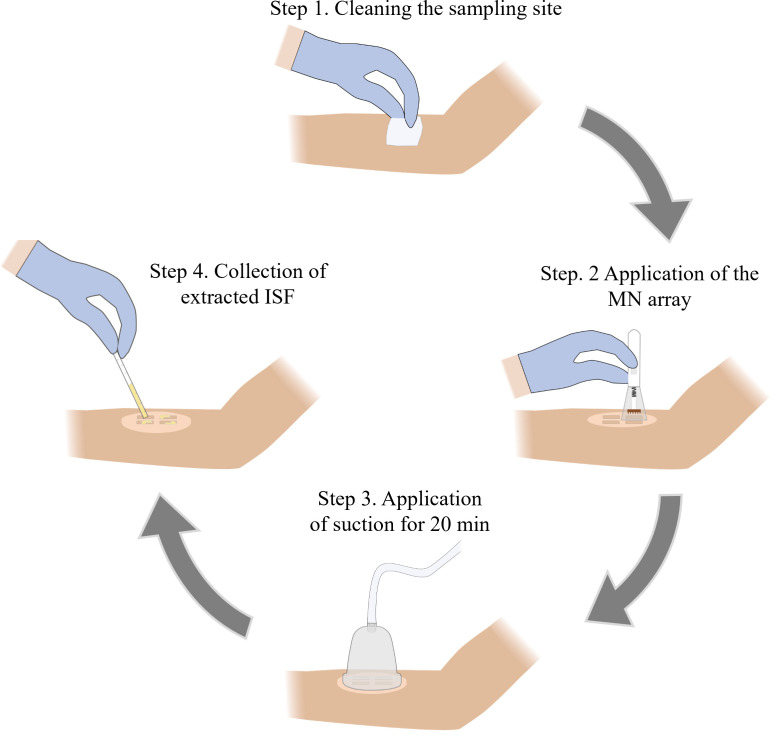


**Microneedle (MN)-based sampling of dermal interstitial fluid (ISF) using a vacuum-assisted skin patch**

## Background

The diagnosis, prognosis, and monitoring of many diseases rely on the detection and/or quantification of biomarkers in blood. While blood sampling is a routine medical procedure, it poses risks of infection and can lead to complications, particularly in newborns and individuals with blood clotting disorders [1,2]. In addition, the discomfort associated with blood sampling can deter individuals with blood or needle phobias from getting tested [3,4]. Interstitial fluid (ISF), which is found in the extracellular space in tissues, has garnered much attention as a diagnostic fluid due to its similar biomarker content to blood [5–9]. Prior studies have shown that ISF collected from skin (dermal ISF) is a promising source for biomarkers (e.g., metabolites, proteins, nucleic acids, and exosomes). However, progress in the use of ISF as a diagnostic fluid is hampered by the lack of rapid and minimally invasive methods for collecting ample quantities of fluid [10,11].

Various methods for extracting ISF from skin have been reported, including the creation of suction blisters [7,8,12], microdialysis [13], open-flow microperfusion [14], laser microporation [15], and reverse iontophoresis [16]. While effective, these methods are time-consuming (~1 h), require the use of specialized equipment, and/or involve invasive procedures that can cause skin erythema and dehydration [7]. Microneedle (MN)-based techniques have also been demonstrated for the collection of human ISF, being faster and less invasive [5,12,17,18]. However, the fluid volumes collected from these techniques are too low (~1–5 μL) for biomolecular analysis using conventional diagnostic assays. For example, lateral flow immunochromatographic assays (LFIAs) require at least ~15 μL of sample, western blot requires ~15–60 μL of sample, and enzyme-linked immunosorbent assay (ELISA) requires 50–100 μL of sample.

We have recently reported a simple and minimally invasive method for sampling larger quantities of ISF from human skin [19]. This approach involves the use of a high-density MN array to generate micropores in the skin followed by the attachment of a rigid skin patch and application of mild vacuum pressure using a portable hand pump. The design of the MN array and the parameters associated with the sample collection process (number of MN insertions and the duration of vacuum application) were optimized in our prior study, and this protocol is based on these optimized parameters. The sampling efficiency of this technique was evaluated by collecting dermal ISF from 28 human volunteers, which yielded an average collection volume of 20.8 μL, a ∼6-fold improvement over existing ISF sampling methods. This technique was well-tolerated and reported as being nearly pain-free by all the volunteers. We envision that this protocol will be useful for physicians, scientists, and bioengineers interested in analyzing ISF for biomarkers that can be used for various diagnostic applications, including disease diagnosis and prognosis, as well as for monitoring therapeutic response.

## Materials and reagents

**Reagents**

1. IP-Q photoresin (Nanoscribe, catalog number: 37071000), store in a UV-protected and airtight container in a cool dry environment away from direct sunlight at 4–8 °C; use only under yellow light with λ > 500 nm

2. SU-8 developer (Kayaku Advanced Materials, catalog number: Y020100)

3. Sylgard 184 silicone elastomer kit (Dow, SKU: 4019862)

4. SU-8 2025 photoresist (Kayaku Advanced Materials, catalog number: Y111069-0500L1GL), store in a UV-protected and airtight container in a cool dry environment away from direct sunlight at 4–21 °C; use only under yellow light with λ > 500 nm

5. Dichloro-p-cyclophane (Parylene C dimer) (Specialty Coating Systems, CAS: 28804-46-8)

6. 2-Propanol (IPA) >99.5% ACS (VWR, catalog number: BDH1133-4LG)

7. Deionized water (17.6 MΩ·cm)

8. Liquid nitrogen

9. SU-8 developer (EMD Performance Materials, AZ® Kwik Strip)

**Solutions**

1. Polydimethylsiloxane (PDMS) (see Recipes)

2. 70% IPA (see Recipes)

**Recipes**

**1. Polydimethylsiloxane (PDMS)**

**Table:** 

Reagent	Final concentration	Quantity or Volume
184 silicone elastomer base	n/a	40 g
184 silicone elastomer curing agent	n/a	4 g
Total	n/a	44 g

**2. 70% isopropyl alcohol (IPA)**

**Table:** 

Reagent	Final concentration	Quantity or Volume
2-Propanol	100%	70 mL
Deionized water	n/a	30 mL
Total	n/a	100 mL

**Laboratory supplies**

1. 3 mm thick clear poly(methyl methacrylate) (PMMA) sheet (McMaster Carr, catalog number: 87225K23)

2. 1.5 mm thick clear PMMA sheet (McMaster Carr, catalog number: 8574K51)

3. Polystyrene Petri dish (VWR, catalog number: 89230-472)

4. Plastic mixing cup (Fisher Scientific, catalog number: S04202)

5. Disposable stirring spatula (Millipore Sigma, catalog number: BR759800-500EA)

6. Razor blade (Bates, model: RZ50)

7. Kimwipes (Fisher Scientific, catalog number: 06-666)

8. Microscope glass slide (Fisher Scientific, catalog number: 125444)

9. Micropipette tips [Fisher Scientific, catalog numbers: 02-707-430 (0–200 μL), 02-707-432 (2–20 μL), 02-707-404 (100–1000 μL)]

10. 50 mL conical centrifuge tubes (Fisher Scientific, catalog number: 339653)

11. Double-sided microfluidic tape (3M Company, model: 9972A)

12. Medical-grade pressured-sensitive adhesive tape (Adhesives Research, model: 90106NB)

13. Alcohol prep pad (Fisher Healthcare, catalog number: 22-363-750)

14. Capillary tube, 70 μL (Fisher Scientific, catalog number: 22-260943)

15. Capillary plunger (CoaguSense, catalog number: 03P52-54)

16. Adhesive remover wipes (Smith & Nephew, catalog number: 402300)

17. 0.5 mL protein low-bind microcentrifuge tubes (Eppendorf, catalog number: 4030-8434)

18. 42 mm diameter vacuum cup and hand pump (Hansol Medical Equipment, model: 2018-01-26-0775)

19. Microneedle spring-loaded applicator (Micropoint Technologies)

20. Tweezers (Fisher Scientific, catalog number: 12-000-127)

21. 250 mL glass beaker (Corning Pyrex, catalog number: 1003-250)

22. Nitrile gloves (VWR, catalog number: 76518-336)

## Equipment

1. Photonic lithography system (Nanoscribe, model: Photonic Professional GT+)

2. Forced air oven (VWR, catalog number: 89511-410)

3. 6 × 50 mL angle rotor and laboratory centrifuge (VWR, catalog numbers: 76181-202, 76181-190)

4. MiniSpin plus mini centrifuge (Eppendorf, catalog number: 022620207)

5. 50 W, 365 nm UV lamp (SUNUV)

6. Parylene deposition system (Specialty Coating Systems, model: PDS 2010 Labcoater)

7. Digital microscope (Keyence Corporation, model: VHX-7000)

8. CO_2_ laser cutter (Universal Laser Systems, model: VLS3.75)

9. Dremel MultiPro rotary tool (Dremel, model: 395)

10. Liquid nitrogen dewar (U.S. Solid, model: USS-LNT00001)

11. Nalgene vacuum chamber (Thermo Scientific, catalog number: 5305-0609)

12. Laboport N 816 pump (Fisher Scientific, catalog number: 13-880-31)

13. Portable precision balance (Ohaus, model: SPX2201)

14. Digital timer (Fisher Scientific, catalog number: 06-664-252)

15. Micropipette (Eppendorf, model: 2231300004)

## Software and datasets

1. NX Student Edition (Siemens Digital Industries Software, Version 1934, 2020)

2. DeScribe (Nanoscribe, Photonic Professional GT+, 2021)

3. AutoCAD (Autodesk, Student version 2021)

4. Prism version 9.5 (GraphPad Software)

## Procedure

**A. Fabrication of the MN array master**

**Figure 1.**
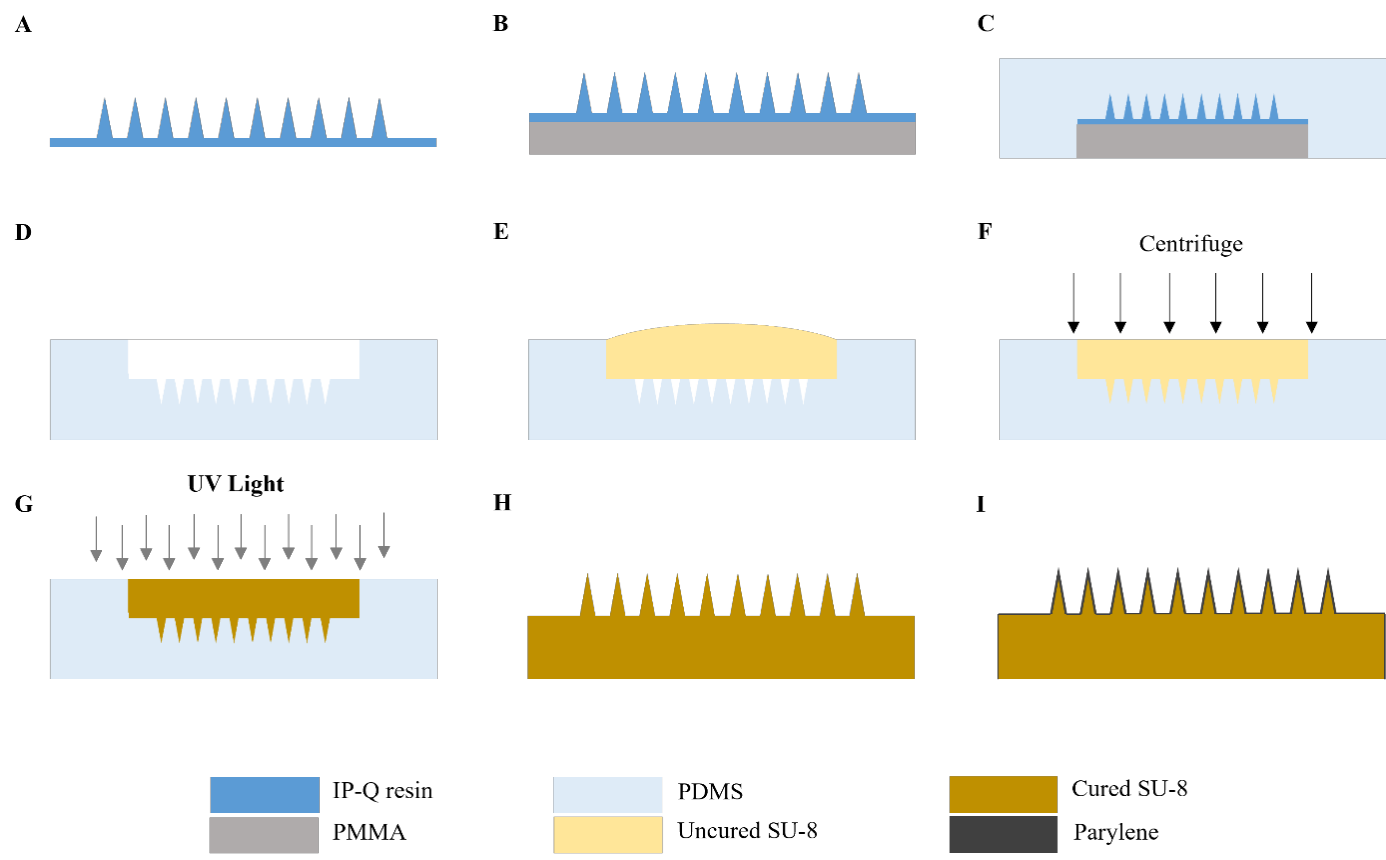
Overview of microneedle (MN) array fabrication procedure. A. The MN array master is fabricated using a Photonic Professional GT lithography system. B. A 3 mm thick PMMA substrate is attached to the backside of the master. C. PDMS is poured over the master and cured in the oven at 80 °C for 2 h. D. The MN master is removed from the PDMS and carefully cut to make the master mold. E. SU-8 photoresist is drop-casted onto the mold. F. Mold is centrifuged at 2,500× *g* for 15 min. G. SU-8-filled mold is exposed to 365 nm UV light for 3 min to cure the photoresist. H. MN array replica is removed from the mold. I. MN array replica is coated with parylene. This figure is adapted with permission from Jiang et al., 2024.

1. Use NX Student Edition software to create the design for the MN array according to the specifications below.

2. Design a MN with a conical shape having a base diameter of 200 μm and height of 450 μm (Figure S1A).

3. Create a 20 × 20 array of MNs with a needle-to-needle spacing of 400 μm on a 10 × 10 mm square base (thickness = 1 mm) (Figure S1B). See Note 1 for alternative MN design considerations.

4. Save the design as a .stl file.

5. Load the .stl file into Nanoscribe DeScribe software and convert the file to a .gwl file.

6. Load the files into a Photonic Professional GT+ lithography system and print the master using IP-Q resin and a 10× lens, following the manufacturer’s recommended procedure (Figure 1A) (Notes 2–3).

7. After printing, fill one glass beaker with 50 mL of SU-8 developer and another glass beaker with 50 mL of 100% IPA. Use tweezers to gently submerge the master in SU-8 developer for approximately 2 min, then submerge the master in 100% IPA for approximately 2 min.

8. Inspect the master using a microscope to ensure that it was printed correctly and does not have any defects or imperfections (e.g., bubbles, cracks, or broken tips) (Troubleshooting Note 1).

9. Use AutoCAD software to create a 10 × 10 mm square to be used as a substrate for the master.

10. Export the drawing to a laser cutter as a .dwg file.

11. Use the laser cutter to cut the substrate from a 3 mm thick PMMA sheet with double-sided adhesive attached to one side (Note 4).

12. Adhere the substrate to the backside of the master (Figure 1B).

**B. Fabrication of the master mold**

1. Place the master in the middle of a Petri dish with the MNs facing upward.

2. Combine 40 g of Sylgard 184 elastomer base with 4 g of curing agent in a mixing cup and stir the mixture vigorously for 2 min using a disposable stir rod.

3. Place the PDMS mixture into a vacuum chamber and open the vacuum valve (vacuum gauge level should go past 15 inch/Hg). Degas for 30 min (most of the air bubbles should be removed from the mixture).

4. Pour the degassed PDMS mixture onto the master in the Petri dish. Ensure the master is completely covered (Figure 1C).

5. Place the Petri dish into a vacuum chamber and open the vacuum valve (vacuum gauge level should go past 15 inch/Hg). Degas for 30 min or until all the air bubbles are removed from the mixture.

6. Place the Petri dish in a convection oven preheated at 80 °C for 2 h to cure the PDMS mixture.

7. Remove the Petri dish from the oven and allow the cured PDMS to cool to room temperature.

8. Use a razor blade to carefully cut out the cured PDMS from the Petri dish.

9. Remove the master from the cured PDMS by slowly peeling it out and cut off excess PDMS to create the master mold (Figure 1D).

10. Inspect the mold using a microscope to ensure that it does not contain any defects or imperfections (e.g., bubbles, voids, or uncured PDMS) (Troubleshooting Note 2).

**C. Fabrication of the MN array replica**

1. Use tweezers to submerge the mold in 70% IPA for 30 min.

2. Remove the mold from the IPA solution and let it air-dry for at least 1 h at room temperature.

3. Place the dried mold on a flat, stable surface and pour SU-8 2025 photoresist into the mold, ensuring the mold cavity is completely filled (Figure 1E).

4. Use tweezers to place the mold into a 50 mL centrifuge tube (Note 5). Ensure the MN tips are oriented toward the bottom of the centrifuge.

5. Centrifuge the tube at 2,500× *g* for 15 min (Figure 1F).

6. Remove the mold from the tube and place it under a UV lamp for 3 min to cure the SU-8 photoresist (Figure 1G).

7. Remove the cured MN array replica from the mold using gloved hands (Figure 1H).

8. Inspect the replica using a microscope to ensure that it does not contain any defects or imperfections (e.g., missing or cracked tips) (Troubleshooting Note 3).

9. Place the MN array replica into a parylene deposition system. Load the system with 1 g of parylene C dimer and run the coating process following the manufacturer’s recommended procedure (Notes 6–7).

10. Remove the parylene-coated MN array from the deposition system and inspect it using a microscope to ensure that the array is completely coated and the coating is uniform (Figure 1I) (Troubleshooting Note 4). See Note 8 for guidance on batch fabricating MN array replicas.

11. The MN array replica(s) can be stored in a Petri dish (MN tips facing upward) at ambient conditions.

**D. Fabrication of skin patch**

1. Use AutoCAD software to create the design for the skin patch according to the specifications below.

2. Design the skin patch as a 42 mm diameter circle with a 2 × 2 array of 11 × 11 mm square cutouts (MN insertion sites) with 2 mm spacing (Figure S2). See Note 1 for alternative skin patch design considerations.

3. Export the drawing to a laser cutter as a .dwg file.

4. Use the laser cutter to cut the patch from a 1.5 mm thick PMMA sheet with double-sided microfluidic tape attached to one side.

5. Use a Dremel rotary tool to smooth the interior edges of the cutouts.

6. Spray the patch with 70% IPA and wipe it clean using a Kimwipe to remove any debris.

7. Use a laser cutter to cut medical-grade adhesive tape with the patch design to generate the skin patch sticker.

**E. ISF sample collection from human volunteers**

**Figure 2.**
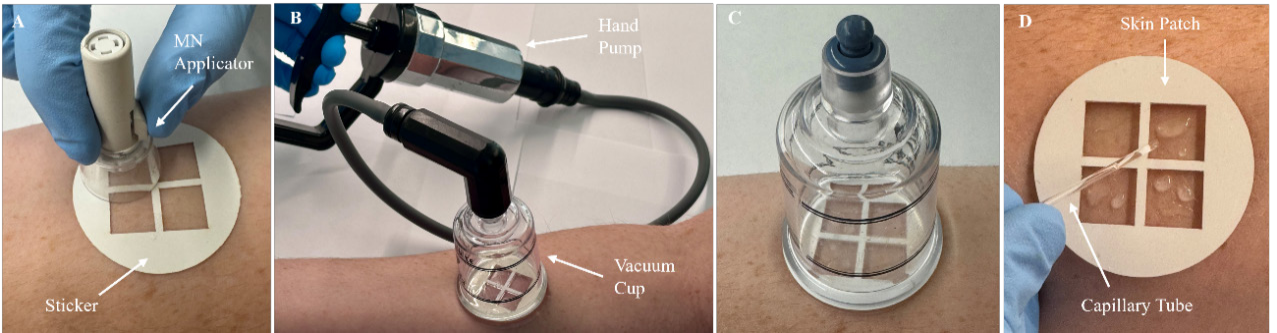
Overview of interstitial fluid (ISF) sampling procedure. A. The sticker is adhered to the anterior forearm, followed by microneedle (MN) insertion using the MN applicator. B. The skin patch is attached to the sticker, followed by the attachment of a vacuum cup. Vacuum pressure is generated in the cup using a hand pump. C. The hand pump is removed from the vacuum cup and vacuum pressure is maintained for 20 min. During this time, extracted ISF can be observed on the skin. D. The vacuum cup is removed and the extracted ISF is collected using a capillary tube.

1. Remove the MN array from the Petri dish and rinse it with 70% IPA followed by air drying at room temperature.

2. Wipe the anterior forearm of the volunteer using a fresh alcohol prep pad and allow it to dry (Note 9).

3. Adhere the skin patch sticker to the cleaned forearm.

4. Load the MN array into an MN applicator following the manufacturer’s instructions.

5. Align the MN array over one of the MN insertion sites and place the MN applicator on the sticker (Figure 2A). Press the applicator button to apply the MN array to the skin.

6. Reload the MN applicator with the same MN array following the manufacturer’s instructions. Repeat step E5 two more times for a total of three MN applications per insertion site.

7. Repeat steps E5–6 using the same MN array at the remaining three insertion sites for a total of 12 MN applications (Note 10).

8. Immediately following the completion of all 12 MN applications, start a timer for 3 min (Note 11).

9. While the timer is counting down, remove the backing from the sticker and adhere the skin patch to it, ensuring that the cutouts are aligned and that the patch is firmly attached to the sticker.

10. While the timer is counting down, remove the backing from the skin patch and adhere the vacuum cup to it, ensuring that they are securely attached with no gaps between the cup and patch.

11. After 3 min have passed, attach the hand pump to the vacuum cup and fully pull the handle back once to generate vacuum pressure inside the cup (Figure 2B) (Troubleshooting Note 5).

12. Remove the hand pump from the vacuum cup and maintain vacuum pressure for 20 min (Note 12) (Troubleshooting Note 5).

13. Observe the cutouts of the skin patch for the formation of droplets of ISF on the skin, which should appear ~5–10 min after vacuum application (Figure 2C) (Troubleshooting Notes 6–7).

14. After 20 min have passed, release the vacuum by pulling the pressure release valve on the top of the vacuum cup and detach the vacuum cup from the skin patch (Note 13).

15. Use a capillary tube to collect the extracted ISF from the skin (Figure 2D).

16. Remove the patch from the volunteer’s forearm by slowly peeling one edge off the skin and using an adhesive remover pad to gradually peel away the rest of the patch.

17. Wipe the sampling site using a fresh alcohol prep pad.

**F. Sample processing and storage**

1. Transfer the collected ISF sample from the capillary tube into a low-bind microcentrifuge tube using a capillary plunger (Note 14).

2. Incubate the sample for 1 h at room temperature to allow any particulates to sediment.

3. Centrifuge the ISF sample at 6,700× *g* for 10 min using a microcentrifuge.

4. Use a micropipette to transfer the supernatant to a new low-bind microcentrifuge tube.

5. Snap-freeze the supernatant by immersing the microcentrifuge tube in liquid nitrogen for 5 min.

6. Immediately store the snap-frozen sample at -80 °C until further analysis (Note 15).

## Data analysis

Data analysis involved in this protocol includes inspecting the fabricated MN array master and replica using microscopy, examining the collected ISF sample via microscopy, and measuring the volume of the collected ISF sample. Briefly, microscopic inspection of the master, replica, and parylene-coated MN array was performed by imaging at 80× magnification. Microscopic examination of all collected ISF samples was performed by imaging the ISF on a glass microscope slide at 200× magnification. The volume of ISF collected from volunteers was determined by aspirating the ISF sample (after being transferred into a low-bind microcentrifuge tube) using a calibrated 2–20 μL micropipette. The collected volume for each volunteer was determined by averaging two independent sample collections. The mean ISF volume collected by this sampling technique was determined by averaging the volumes of samples collected from 28 volunteers. Means, standard deviations, and statistical analyses were conducted using Prism version 9.5.

## Validation of protocol

This protocol has been used and validated in the following research article:

- Jiang et al. [19]. Microneedle-based sampling of dermal interstitial fluid using a vacuum-assisted skin patch. Cell Reports Physical Science.

**Figure 3.**
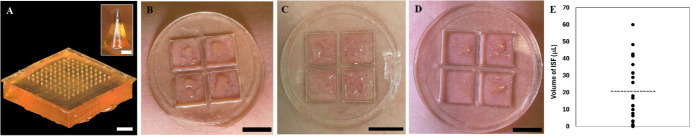
Validation of microneedle (MN) array fabrication and interstitial fluid (ISF) sample collection. A. Optical micrograph of the MN array. Scale bar, 1,000 μm. Inset shows a close-up view of a single MN. Scale bar, 100 μm. B–D. Photographs of ISF collected on three different volunteers. Scale bars, 10 mm. E. Mean volumes of dermal ISF collected from the 28 volunteers. Each data point represents the average from two independent sample collections from a single volunteer. The horizontal dotted line represents the average volume of all sample collections, n = 28. Panels B–C are adapted with permission from Jiang et al. [19].

The experimental procedure for fabricating the MN array via centrifugation-assisted replica molding is presented in Video S1, which results in the creation of mechanically robust and sharp MNs (Figure 3A). The strength of the MN array was validated through mechanical testing, as reported by Jiang et al. [19]. Force-displacement curves were generated for MN array replicas with different MN designs and array sizes, including the optimized MN design (base diameter = 200 μm, height = 450 μm) and array size (20 × 20) used in this protocol. This MN array can withstand up to 50 N of compression force, which is 1.5-fold larger than the force required to penetrate human skin, without exhibiting any signs of deformation ([19], Figure S1D). The penetration performance of the MNs was evaluated by inserting the MN array into porcine skin and performing histological analysis on skin sections, which revealed the formation of conical micropores in the epidermis ([19], Figure 2C). Optical micrographs of the MN array after 12 skin insertions revealed that the MNs exhibited no discernible deformation or damage ([19], Figure S3D), validating the ability of the MN array to repeatedly penetrate human skin and generate micropores without fracturing or breaking.

The ISF extraction efficiency was validated by sampling dermal ISF from 28 human volunteers, as reported by Jiang et al., [19]. ISF extracted using this technique was clear to light yellowish in color and generally more viscous than sweat (Figure 3B-D). The average volume of ISF collected from all 28 volunteers was 20.8 ± 19.4 μL (mean ± SD) (Figure 3E). Studies evaluating the safety and tolerability of this technique revealed that it was well tolerated by all volunteers with only minor adverse effects (e.g., skin redness, mild swelling, or slight tenderness localized within the skin patch) that completely resolved within 1 day ([19], Figure S8). Furthermore, volunteers rated the sampling technique as being nearly pain-free ([19], 2024, Figure S7), making it potentially more acceptable to individuals with needle and blood phobias.

Proteomic analysis of dermal ISF collected from five volunteers using this technique was performed using nanoflow liquid chromatography–tandem mass spectrometry, as reported by Jiang et al. [19]. This analysis resulted in the identification of 2,006 distinct proteins in ISF. Of these proteins identified, 610 are associated with diseases as determined by two online biomarker databases (OncoMX [20] and BIONDA [21]), where 98 are also classified in the NCI Early Detection Research Network biomarker database, and five are approved biomarkers by the US Food and Drug Administration. Dermal ISF samples from COVID-19 vaccinees were analyzed for the presence of SARS-CoV-2 neutralizing antibodies using two commercial SARS-CoV-2 neutralizing antibody tests. SARS-CoV-2 neutralization antibodies were detected in dermal ISF from all vaccinees using both assays ([19], Figure 5C–D). These collective results validate dermal ISF as a source of medically relevant protein biomarkers and demonstrate its utility as a diagnostic fluid.

## General notes and troubleshooting

**General notes**

1. Variations in the design of the MNs and array size can be considered and may prove to be beneficial. For example, a different needle height (e.g., 600 μm, 750 μm) or array size (e.g., 10 × 10) may be beneficial for sampling ISF from other parts of the body with different skin thicknesses and/or topography. Modifications to the design of the skin patch (e.g., smaller cutouts, different cutout layout) may be needed depending on the modified MN array design.

2. Special training and cleanroom environments may be required for the use of the Nanoscribe Photonic Professional GT+ lithography system. Alternative 3D printing platforms with high resolution may be suitable to produce similar results.

3. The standard protocol from the manufacturer’s user guide of this model was used without alterations.

4. Special training may be required for the use of a CO_2_ laser cutter.

5. A custom centrifuge tube adapter can be utilized to help stabilize and position the MN array mold(s) in the proper orientation during centrifugation. The tube adapter can be customized based on the specific centrifuge tube used and the angle of the tube holder within the centrifuge rotor. A schematic of the 3D-printed tube adapter used in this procedure is shown in Figure S3.

6. Special training and cleanroom environments may be required for the use of the parylene deposition system.

7. The manufacturer’s recommendations were followed for the procedures. The manufacturer-provided ratio is 1 g of parylene C dimer = 1.5 μm of coating.

8. MN arrays can be batch-fabricated by creating multiple molds and performing replica molding processes in parallel. For example, multiple molds can be centrifuged at once, and multiple replicas can be placed in the parylene deposition system for parylene coating.

9. Research activities involving human subjects must be reviewed and approved by an Institutional Review Board.

10. MN arrays should be discarded at the completion of a sample collection procedure and handled as sharps waste.

11. A 3-min wait period between MN insertion and vacuum application is recommended to prevent the collection of blood with ISF. A longer wait period can be used, although it was observed that waiting >5 min resulted in the collection of a negligible amount of fluid.

12. Use caution when removing the hand pump from the vacuum cup to ensure that the pressure inside the vacuum cup is not released by accidentally pulling on the release valve located on top of the cup.

13. The vacuum cup can be reused after sterilization by placing it in boiling water for 30 min.

14. The sample can be examined via microscopy to detect the presence of blood cells, which would indicate blood contamination.

15. In section F (Sample processing and storage), steps 2–6 are optional for applications where the sample is to be analyzed without processing (e.g., rapid diagnostic testing using a lateral flow immunoassay).

**Troubleshooting**

Problem 1: Defects in the MN array master.

Possible cause: There were issues with resin preparation and/or the 3D printing process.

Solution: Allow the IP-Q resin to come to room temperature before usage. Ensure there are no bubbles when the resin is applied to the lens for 3D printing. Alter the printing specifications according to the manufacturer’s recommendations.

Problem 2: Defects in the master mold.

Possible cause: There are voids and/or bubbles, or PDMS is not fully cured.

Solution: Degas the PDMS for a longer amount of time in the vacuum chamber. Leave the PDMS in the oven for a longer period of time.

Problem 3: Inconsistent formation of MN tips.

Possible cause: SU-8 photoresist spilled out of the mold during centrifugation.

Solution: Place a piece of plastic film over the mold after filling it with SU-8 photoresist. Create a conical centrifuge tube adapter that allows the mold to be oriented at a fixed angle parallel to the bench during centrifugation.

Problem 4: Defects in the parylene coating.

Possible cause: The parylene layer is too thin to evenly coat the MNs.

Solution: Increase the amount of parylene C dimer. Alter the deposition parameters according to the manufacturer’s recommendations.

Problem 5: Vacuum pressure is not generated in the vacuum cup or cannot be maintained for 20 min.

Possible cause: Several factors could affect the vacuum seal, including gaps or air bubbles within the adhesive layer or excessive hair on the skin.

Solution: Remove and replace the sticker and/or the skin patch. Ensure the sticker/skin patch/vacuum cup are securely attached together with no gaps or air bubbles within the adhesive. Adhere the sticker to a location on the anterior forearm with minimal hair.

Problem 6: No ISF is extracted after 10–15 min.

Possible cause: There were issues with the MN application process. Differences in individuals’ skin, such as topology, thickness, or elasticity, can affect the MN penetration depth and micropore size, inhibiting ISF extraction.

Solution: Remove the vacuum cup and repeat the sampling procedure at the same location or at a different location (e.g., the other arm). It is not recommended to repeat the sampling procedure more than twice at the same location during a single sample collection session. Intrasubject and intersubject variability in the amount of ISF collected using this protocol is to be expected.

Problem 7: Blood dots are formed on the skin.

Possible cause: Vacuum is applied too quickly following MN insertion, or the vacuum pressure is too strong.

Solution: Increase the wait time between MN insertion and vacuum application to 4–5 min. Use a weaker vacuum pressure by pulling the pump handle halfway to three-quarters of the way back.

## Supplementary information

The following supporting information can be downloaded here.

Figure S1. Designs of the MN and MN array

Figure S2. Design of the skin patch

Figure S3. Design of the centrifuge tube adapter

Video S1. Experimental procedure for fabricating the MN array via centrifugation-assisted replica molding

## References

[1] Bogers J. P., Bui H., Herruer M. and Cohen D. (2015). Capillary compared to venous blood sampling in clozapine treatment: patients׳ and healthcare practitioners׳ experiences with a point-of-care device. Eur Neuropsychopharmacol. 25(3): 319 324 324. 10.1016/j.euroneuro.2014.11.022 25548103

[2] Lassandro G., Amoruso A., Palladino V., Palmieri V. V. and Giordano P. (2021). The Risk of Venipuncture in Newborn with Severe Hemophilia: Case Report of a Large Elbow Hemorrhage and Literature Review of Compartment Syndrome. Hematol Rep. 13(2): 8967 10.4081/hr.2021.8967 34221293 PMC8215530

[3] McMurtry C. M., Pillai Riddell R., Taddio A., Racine N., Asmundson G. J. G., Noel M., Chambers C. T. and Shah V. (2015). Far From“Just a Poke”. Clin J Pain. 31: S3–S11. https://doi.org/10.1097/ajp.0000000000000272 PMC4900413 26352920

[4] Öst L. G. (1992). Blood and injection phobia: Background and cognitive, physiological, and behavioral variables. J Abnorm Psychol. 101(1): 68 74 74. 10.1037//0021-843x.101.1.68 1537975

[5] Samant P. P. and Prausnitz M. R. (2018). Mechanisms of sampling interstitial fluid from skin using a microneedle patch. Proc Natl Acad Sci USA. 115(18): 4583 4588 4588. 10.1073/pnas.1716772115 29666252 PMC5939066

[6] Tran B. Q., Miller P. R., Taylor R. M., Boyd G., Mach P. M., Rosenzweig C. N., Baca J. T., Polsky R. and Glaros T. (2017). Proteomic Characterization of Dermal Interstitial Fluid Extracted Using a Novel Microneedle-Assisted Technique. J Proteome Res. 17(1): 479 485 485. 10.1021/acs.jproteome.7b00642 29172549

[7] Müller A. C., Breitwieser F. P., Fischer H., Schuster C., Brandt O., Colinge J., Superti-Furga G., Stingl G., Elbe-Bürger A., Bennett K. L., .(2012). A Comparative Proteomic Study of Human Skin Suction Blister Fluid from Healthy Individuals Using Immunodepletion and iTRAQ Labeling. J Proteome Res. 11(7): 3715 3727 3727. 10.1021/pr3002035 22578099

[8] Kool J., Reubsaet L., Wesseldijk F., Maravilha R. T., Pinkse M. W., D'Santos C. S., van Hilten J. J., Zijlstra F. J. and Heck A. J. R. (2007). Suction blister fluid as potential body fluid for biomarker proteins. Proteomics. 7(20): 3638 3650 3650. 10.1002/pmic.200600938 17890648

[9] Ribet F., Bendes A., Fredolini C., Dobielewski M., Böttcher M., Beck O., Schwenk J. M., Stemme G. and Roxhed N. (2023). Microneedle Patch for Painless Intradermal Collection of Interstitial Fluid Enabling Multianalyte Measurement of Small Molecules, SARS‐CoV‐2 Antibodies, and Protein Profiling. Adv Healthcare Mater. 12(13): e202202564. https://doi.org/10.1002/adhm.202202564 PMC11468663 36748807

[10] Friedel M., Thompson I. A. P., Kasting G., Polsky R., Cunningham D., Soh H. T. and Heikenfeld J. (2023). Opportunities and challenges in the diagnostic utility of dermal interstitial fluid. Nat Biomed Eng. 7(12): 1541 1555 1555. 10.1038/s41551-022-00998-9 36658344

[11] Saifullah K. M. and Faraji Rad Z. (2023). Sampling Dermal Interstitial Fluid Using Microneedles: A Review of Recent Developments in Sampling Methods and Microneedle‐Based Biosensors. Adv Mater Interfaces. 10(10): e202201763. https://doi.org/10.1002/admi.202201763

[12] Samant P. P., Niedzwiecki M. M., Raviele N., Tran V., Mena-Lapaix J., Walker D. I., Felner E. I., Jones D. P., Miller G. W., Prausnitz M. R., .(2020). Sampling interstitial fluid from human skin using a microneedle patch. Sci Transl Med. 12(571): eaaw0285. https://doi.org/10.1126/scitranslmed.aaw0285 PMC7871333 33239384

[13] KROGSTAD A. L., JANSSON P. A., GISSLÉN P. and LÖNNROTH P. (1996). Microdialysis methodology for the measurement of dermal interstitial fluid in humans. Br J Dermatol. 134(6): 1005 1012 1012. 10.1111/j.1365-2133.1996.tb07934.x 8763416

[14] Schaupp L., Ellmerer M., Brunner G. A., Wutte A., Sendlhofer G., Trajanoski Z., Skrabal F., Pieber T. R. and Wach P. (1999). Direct access to interstitial fluid in adipose tissue in humans by use of open-flow microperfusion. Am J Physiol Endocrinol Metab. 276(2): E401–E408. 10.1152/ajpendo.1999.276.2.e401 9950802

[15] Venugopal M., Feuvrel K. E., Mongin D., Bambot S., Faupel M., Panangadan A., Talukder A. and Pidva R. (2008). Clinical Evaluation of a Novel Interstitial Fluid Sensor System for Remote Continuous Alcohol Monitoring. IEEE Sens J. 8(1): 71 80 80. 10.1109/jsen.2007.912544

[16] Sieg A., Guy R. H. and Delgado-Charro M. B. (2004). Noninvasive Glucose Monitoring by Reverse Iontophoresis in Vivo: Application of the Internal Standard Concept. Clin Chem. 50(8): 1383 1390 1390. 10.1373/clinchem.2004.032862 15155544

[17] Al-Kasasbeh R., Brady A. J., Courtenay A. J., Larrañeta E., McCrudden M. T., D. O’Kane, Liggett S. and Donnelly R. F. (2020). Evaluation of the clinical impact of repeat application of hydrogel-forming microneedle array patches. Drug Deliv Transl Res. 10(3): 690 705 705. 10.1007/s13346-020-00727-2 32103450 PMC7228965

[18] Mukerjee E., Collins S., Isseroff R. and Smith R. (2004). Microneedle array for transdermal biological fluid extraction and in situ analysis. Sens Actuators, A. 114: 267 275 275. 10.1016/j.sna.2003.11.008

[19] Jiang X., Wilkirson E. C., Bailey A. O., Russell W. K. and Lillehoj P. B. (2024). Microneedle-based sampling of dermal interstitial fluid using a vacuum-assisted skin patch. Cell Rep Phys Sci. 5(6): 101975 10.1016/j.xcrp.2024.101975 38947182 PMC11211974

[20] Dingerdissen H. M., Bastian F., Vijay-Shanker K., Robinson-Rechavi M., Bell A., Gogate N., Gupta S., Holmes E., Kahsay R., Keeney J., .(2020). OncoMX: A Knowledgebase for Exploring Cancer Biomarkers in the Context of Related Cancer and Healthy Data. JCO Clin Cancer Inf. 210–220. 10.1200/cci.19.00117 PMC7101249 32142370

[21] Turewicz M., Frericks-Zipper A., Stepath M., Schork K., Ramesh S., Marcus K. and Eisenacher M. (2021). BIONDA: a free database for a fast information on published biomarkers. Bioinf Adv. 1(1): e1093/bioadv/vbab015. 10.1093/bioadv/vbab015 PMC9710600 36700097

